# The Ground State of Monolayer Graphene in a Strong Magnetic Field

**DOI:** 10.1038/srep22423

**Published:** 2016-03-01

**Authors:** Lian-Ao Wu, Mike Guidry

**Affiliations:** 1 IKERBASQUE, Basque Foundation for Science, 48011 Bilbao, Spain; 2 Department of Theoretical Physics and History of Science, Basque Country University (EHU/UPV), Post Office Box 644, 48080 Bilbao, Spain; 3 Department of Physics and Astronomy, University of Tennessee, Knoxville, Tennessee 37996, USA

## Abstract

Experiments indicate that the ground state of graphene in a strong magnetic field exhibits spontaneous breaking of SU(4) symmetry. However, the nature of the corresponding emergent state is unclear because existing theoretical methods approximate the broken-symmetry solutions, yielding nearly-degenerate candidate ground states having different emergent orders. Resolving this ambiguity in the nature of the strong-field ground state is highly desirable, given the importance of graphene for both fundamental physics and technical applications. We have discovered a new SO(8) symmetry that recovers standard graphene SU(4) quantum Hall physics, but predicts two new broken-SU(4) phases and new properties for potential ground states. Our solutions are analytical; thus we capture the essential physics of spontaneously-broken SU(4) states in a powerful yet solvable model useful both in correlating existing data and in suggesting new experiments.

Graphene in a strong magnetic field has approximate SU(4) symmetry^1^,^2^,^3^,^4^,^5^,^6^,^7^,^8^, which permits examining explicit symmetry breaking by small terms in the Hamiltonian. However, the ground state is strongly insulating, with rapid divergence of longitudinal resistance at a critical magnetic field *B*_c_ 9. The dependence of *B*_c_ on sample impurities suggests that the resistance is an intrinsic property of an *emergent state* differing qualitatively from perturbed SU(4) solutions (spontaneous symmetry breaking)^10^. SU(4) symmetry can suggest the form of possible emergent states but cannot describe them quantitatively. Numerical simulations find various possible ground states having similar energies but differing structure. Thus the nature of the insulating ground state remains elusive.

Here we show that SU(4) symmetry can be extended to an SO(8) symmetry that recovers graphene SU(4) physics, but that implies new low-energy modes that transcend SU(4) symmetry and for which solutions may be obtained *analytically.* As a first application we revisit the nature of the ground state for undoped monolayer graphene in a magnetic field.

## Results

### A General Hamiltonian

Good reviews of graphene physics are available^8^,^11^,^12^; we recall here only features relevant for the present discussion. Graphene is bipartite with sublattices A and B; the quantity specifying whether an electron is on the A or B sublattice is termed the *sublattice pseudospin*. The dispersion computed in tight-binding approximation^11^,^12^ indicates two inequivalent sets of points in the Brillouin zone, labeled *K* and *K*′. The two-fold K degree of freedom is termed *valley isospin*. Near these K-points the dispersion is linear, leading to *Dirac cones.* For undoped graphene the Fermi surface lies at the apex of the cones, where the level density vanishes and the effective electronic mass tends to zero. Hence, near the K points low-energy electrons for undoped graphene in zero magnetic field obey a massless Dirac equation and behave mathematically as *massless chiral fermions,* with chirality related to projection of the sublattice pseudospin.

In a magnetic field the massless Dirac equation may be solved with an appropriate vector potential and the resulting Landau levels (LLs) are labeled by integers. The *n* = 0 level is unusual in that it is half filled in the ground state of undoped graphene, leading to the anomalous counting observed in the graphene quantum Hall effect^13^,^14^. For low-energy excitations in each valley (*K* or *K*′), inter-valley tunneling may be ignored and the electrons in the valley reside entirely on either the A or B sublattice, implying that for the *n* = 0 LL valley isospin and sublattice pseudospin are equivalent labels. We shall be concerned primarily with this *n* = 0 Landau level, which has, in addition to the Landau orbital degeneracy, a 4-fold degeneracy corresponding to spin and valley isospin.

The largest energy scales are the LL separation and Coulomb energy. For neutral graphene the LL separation is approximately three times larger than the Coulomb energy, which is in turn much larger than other interactions. Hence, we shall ignore inter-LL excitations and consider only a single *n* = 0 LL. Justification and caveats for this approximation are discussed in ref. 5. We adopt a Hamiltonian^5^,^6^


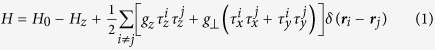


where the Pauli matrices *τ*_*α*_ operate on valley isospin, the Pauli matrices *σ*_*α*_ operate on electronic spin, *g*_*z*_ and *g*_⊥_ are coupling constants, *μ*_B_ is the Bohr magneton, and the spin *z* direction is assumed aligned with the magnetic field. The three terms in Eq. (1) represent the valley-independent Coulomb interaction, the Zeeman energy, and the short-range valley-dependent interactions, respectively.

**Figure 1:**
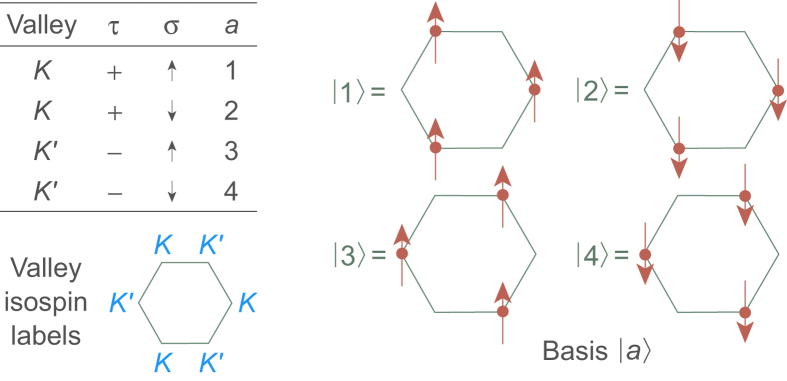
Isospin–spin quantum numbers and basis vectors.

The four internal states representing possible combinations of the projection of the spin *σ* and the projection of the valley isospin *τ* are displayed in Fig. 1. Symmetries of the Hamiltonian (1) may be investigated by introducing the 15 operators


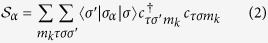



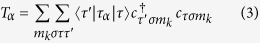



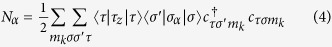



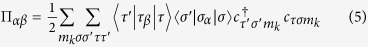


where *c*^†^(*c*) create (annihilate) fermions, 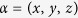, 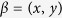, *τ* and *σ* are defined in Fig. 1, and *m*_*k*_ labels orbitally-degenerate LL states. Physically, 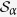 is total spin, *T*_*α*_ is total valley isospin, *N*_*α*_ is a Néel vector measuring the difference in spins on the A and B sublattices, and the Π_*αβ*_ couple spin and valley isospin. Under commutation the operators (2–5) close an SU(4) algebra that commutes with the Coulomb interaction^5^,^6^. If terms 2 and 3 in Eq. (1) are small compared with the first, the Hamiltonian has approximate SU(4) invariance. Explicit breaking of SU(4) depends on the values of *g*_*z*_ and *g*_⊥_. Four symmetry-breaking patterns have been discussed^5^,^6^. Alternative approaches to the graphene problem have addressed some of the same issues discussed here (for example, refs 15, 16, 17) but our emphasis will be on approaches based on extensions of SU(4) symmetry.

### Generators of our model

For a 2-*N* dimensional fermionic space the most general bilinear products 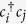 of creation–annihilation operators and their hermitian conjugates generate an SU(2*N*) Lie algebra under commutation. Adding the most general pair operators 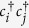 and *c*_*i*_*c*_*j*_ extends SU(2*N*) to SO(4*N*)^18^,^19^. The extended symmetry permits defining a (collective) subspace of the full Hilbert space spanned by products of pair creation operators acting on the pair vacuum. An effective Hamiltonian constructed from a polynomial in the Casimir invariants of all groups in the subgroup chains of SO(4*N*) will then represent the most general Hamiltonian for the collective subspace, and will be diagonal in the subspace basis for each dynamical symmetry chain. Thus, the manybody problem can be *solved exactly* in the symmetry limits defined by each subgroup chain^20^, and analytically in coherent-state approximation^21^,^22^,^23^,^24^,^25^ otherwise. This approach has been applied extensively to strongly-correlated fermions in various fields; for representative examples see^20^,^26^,^27^,^28^,^29^.

For graphene we assume a single *n* = 0 LL with creation operators 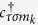 and hermitian conjugates 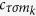. Degeneracy of the LL is denoted by 2Ω. Accounting for 4-fold spin–valley degeneracy, 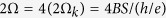, where 2Ω_*k*_ is the LL orbital degeneracy, *B* is magnetic field, and *S* sample size. The *fractional occupation* of the LL is 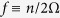, where *n* is electron number, and the *filling factor* is 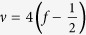.

Now we add to the 15 SU(4) generators of Eqs (2, 3, 4, 5) the charge operator 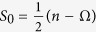, the 6 pairing operators *S*^†^ and 
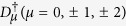
, and their 6 hermitian conjugates, with


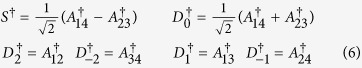


where 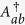 creates a pair of electrons, one in the *a* = (*τ*_1_, *σ*_1_) level and one in the *b* = (*τ*_2_, *σ*_2_) level, with the total *m*_*k*_ of each pair coupled to zero term by term:


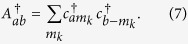


We also introduce for later use the linear combinations


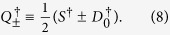


**Figure 2:**
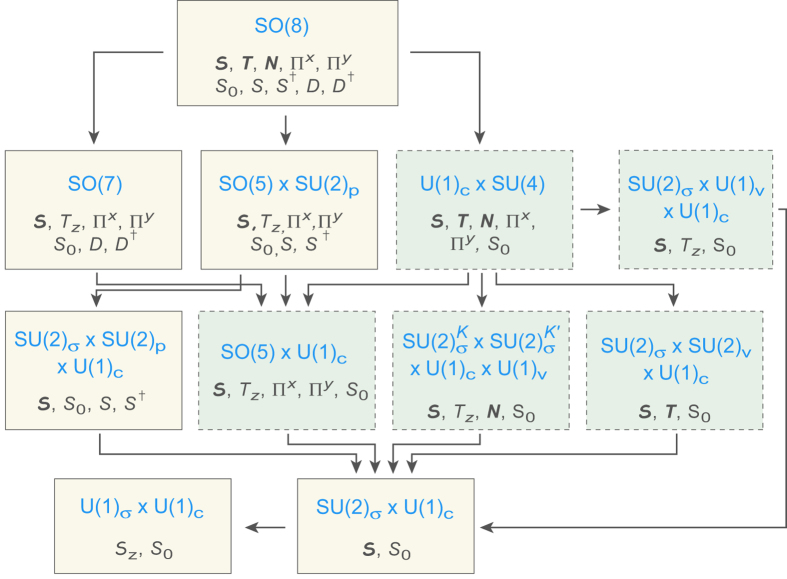
SO(8) subgroup chains with group generators. Dashed boundaries and darker shading indicate subgroups defining the SU(4) quantum Hall model. We see that SO(8) subsumes graphene SU(4) but has a richer structure with additional subgroup chains.

The 28 operators 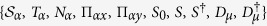 generate an SO(8) algebra with a graphene SU(4) subalgebra. The full structure for SO(8) subgroup chains is given in Fig. 2.

**Figure 3:**
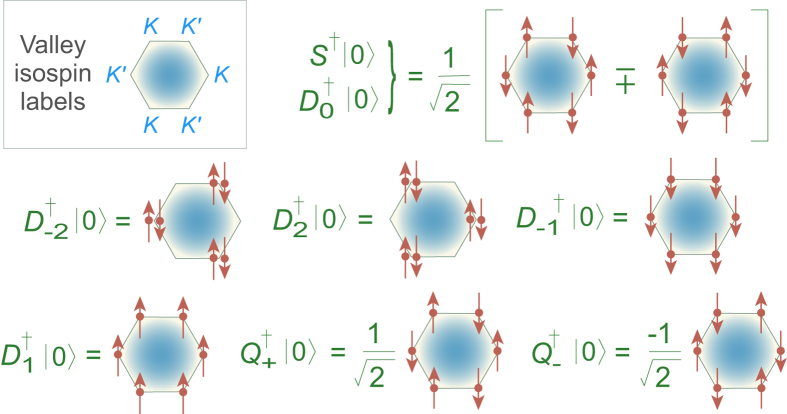
Configurations created by the operators of Eqs (6 and 8) operating on the pair vacuum 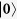. Location of the dots (*K* or *K*′ site) indicates the valley isospin; arrows indicate the spin polarization.

Pair configurations created by generators of Eqs (6 and 8) operating on the pair vacuum are given in Fig. 3. Kharitonov^5^ has classified collective modes for the *n* = 0 LL using pairs similar physically to ours: 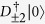 creates spin-singlet charge density waves (CDW), 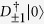 creates ferromagnetic (FM) states, and 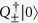 creates antiferromagnetic (AF) states. These states may be classified according to 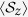, which measures net spin and characterizes *FM order,* 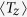, which measures the sublattice charge difference and characterizes *CDW order,* and 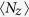, which measures the sublattice spin difference and characterizes *AF order.* Thus pairs in Eqs (6, 7, 8) define modes already discussed^5^,^6^, but now SO(8) symmetry permits *analytical solutions* for corresponding collective modes.

### General wavefunction

Broken-symmetry states based on graphene SU(4) have been expressed in terms of the pair wavefunction^5^


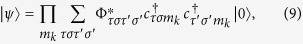


where the product is over the LL orbital degeneracy label *m*_*k*_ and the sum is over spin and valley labels. Now let us consider SO(8) pairs. All states of an irreducible representation may be constructed by successive application of raising and lowering operators to a highest-weight (HW) state (Cartan–Dynkin method)^19^. Let *u* denote the number of broken pairs. For *u* = 0 states at half filling the pair number is 
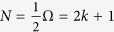
 and the U(4) representation is 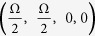. We choose the HW state as the pair state that results from placing one electron in the *a* = 1 and one in the *a* = 2 basis states (see Fig. 1),


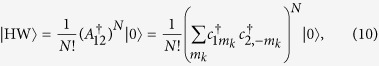


where the sum runs over the *N* states in the LL labeled by the 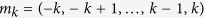 orbital quantum number. Writing the sum over *m*_*k*_ in Eq. (10) out explicitly and invoking antisymmetry eliminates most terms and leaves


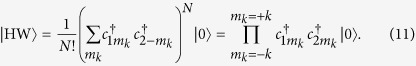


Thus the SO(8) *u* = 0 HW state is *equivalent to a product of pairs*, one for each *m*_*k*_ in the LL.

Other states can be constructed by applying successively to 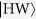 ladder operators that are functions of the generators 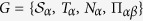 of Eqs (2, 3, 4, 5). For for an arbitrary state 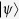 in the weight space 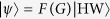, where *F*(*G*) is specified by the Cartan–Dynkin procedure. For example, applying the isospin lowering operator 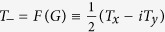 gives


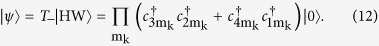


Likewise, all other states of the *u* = 0 representation can be constructed by using successive applications of raising and lowering operators fashioned from the generators of Eqs (2, 3, 4, 5), and they will take the product of sums form (12), just as for Eq. (9). Hence the 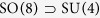 symmetry introduced here recovers existing understanding^5^,^6^ of states expected from spontaneous breaking of SU(4) by short-range correlations.

### Group chains

Figure 2 contains 7 subgroup chains. Each defines a dynamical symmetry realized for specific choices of the SO(8) Hamiltonian parameters, and yields an *exact manybody solution* using standard techniques. We shall deal with these exact solutions in future papers. Here, we interpret the states implied by Fig. 2 using coherent state (CS) approximations^25^. The full coherent state solution will be presented in a later paper, but we illustrate here for subgroup chains containing SO(5) in Fig. 2, corresponding to solutions that are linear combinations of the symmetry-limit solutions for the 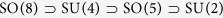, the 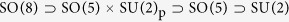, and the 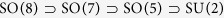 dynamical symmetries. The required group theory is already known^20^,^25^,^30^,^31^,^32^,^33^,^34^, so we can (with suitable change of notation and basis) simply transcribe many equations and reinterpret them in terms of graphene physics. Details follow in a later paper but we show here results central to this paper.

#### Energy surfaces

The energy *E*_g_(*n*, *β*) depends only on *n* and a *single order parameter β*. For the symmetry groups g


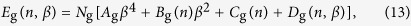


where the group-dependent coefficients are tabulated in ref. 32. This energy surface results from minimizing


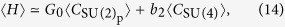


where 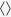 is taken in the CS, *C*_*g*_ denotes the quadratic Casimir invariant of g, and *b*_2_ and *G*_0_ are coupling strengths.

#### Order Parameter

The order parameter *β* distinguishes the phases associated with the subgroup chains in Fig. 2 that contain SO(5). *β* measures AF, since it is related to the AF order parameter 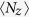 by


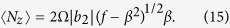


#### Fluctuations

Coherent states violate translational, rotational, and gauge invariance. However, for realistic fields and sample sizes these violations are negligible, yielding a Ginzburg–Landau type theory with microscopic pedigree.

#### Wavefunctions

Closed forms are given for the SO(8) CS wavefunctions in ref. 32. Evaluating these expressions in the 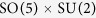 and SU(4) limits, respectively, gives


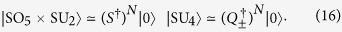


Thus the SU(4) state is a superposition of *Q*_±_ pairs, each with vanishing 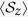 and 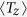 but finite AF order 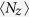. Conversely, the 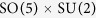 state is a superposition of *S* pairs, each with vanishing 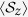, 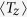, and 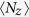. The critical SO(7) state is realized in the transition from 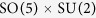 to SU(4) and represents a complex mixture of these wavefunctions.

**Figure 4:**
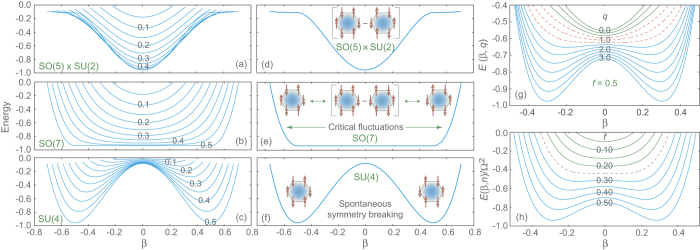
(**a**–**c**) Coherent state energy surfaces as a function of AF order *β* for three SO(8) dynamical symmetry limits. Curves labeled by fractional occupation *f* = *n*/2Ω (particles or holes, since the SO(8) theory is particle–hole symmetric). (**d**–**f**) Ground-state energy for three symmetry limits. Diagrams indicate the wavefunctions suggested by Eq. (16). (**g**) Energy as a function of coupling strength ratio 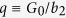 for *f* = 0.5. Solid green curves (*q* ~ 0 − 0.5) indicate 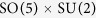 symmetry, solid blue curves for *q* ≥ 1.5 indicate SU(4) symmetry. Dashed red curves for *q* ~ 1 correspond to the critical SO(7) symmetry mediating the quantum phase transition from 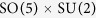 to SU(4). (**h**) Energy surfaces for different occupation fractions *f* at fixed *G*_0_ and *b*_2_ (*q* = 2.5). Solid green curves for *f* ~ 0 − 0.2 indicate 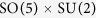 symmetry, Solid blue curves for *f* ~ 0.3 − 0.5 indicate SU(4) symmetry. Curves near *f* ~ 0.25 (dashed red) correspond to SO(7) symmetry mediating the 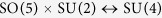 quantum phase transition.

Energies for the 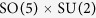, SO(7), and SU(4) limits are shown for several values of *f* = *n*/2Ω in Fig. 4(a–c). The solutions are distinguished by the AF order parameter *β* at the minimum, which is zero for 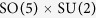, non-zero for SU(4), and indeterminate in the SO(7) critical dynamical symmetry that interpolates between 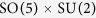 and SU(4) through fluctuations in AF order. For undoped graphene the ground state corresponds to the *f* = 0.5 curves. These are shown in Fig. 4(d–f) for the three symmetry limits, along with a physical interpretation of the states in terms of the wavefunctions (16). Thus the SO(8) dynamical symmetry limits illustrated in Fig. 4(d–f) represent a rich set of collective states that can be distinguished by the expectation value and fluctuations associated with the order parameter *β*.

Quantum phase transitions between symmetry limits may be studied by varying coupling. We rewrite Eq. (14) in terms of a parameter 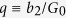, which favors 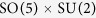 when 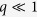, SU(4) when 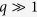, and SO(7) when *q* ~ 1, since


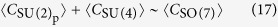


implies SO(7) symmetry if *G*_0_ ~ *b*_2_. Variation of ground state energy with *q* is shown in Fig. 4(g). Alternatively, at fixed *q* phase transitions may be initiated by changing particle occupancy. Figure 4(h) displays a transition from 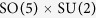 with *β* = 0, through a critical SO(7) symmetry with energy highly degenerate in *β*, to SU(4) with *β* ≠ 0, as *f* is changed at constant *q*.

Thus SO(8) describes analytically a host of broken-SU(4) candidates for the states in graphene being unraveled in modern experiments^7^,^35^,^36^,^37^. These solutions provide a spectrum of excited states as well as ground states. We shall not discuss that here, except to note that all ground state solutions have a gap to electronic and collective excitations. The general theory to be discussed in forthcoming papers can accommodate FM, CDW, and AF states, but for dynamical symmetries containing SO(5) all solutions may be classified by a *single parameter β* that measures AF order: SU(4) states have finite *β* and AF order, but no CDW or FM order, 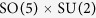 states have *β* = 0 and no AF, CDW, or FM order, and SO(7) states define a critical dynamical symmetry that interpolates between SU(4) and 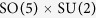 with no AF order but large AF fluctuations, and with no CDW or FM order. We have neglected Zeeman coupling here but it is expected to be small for the *n* = 0 LL^7^, primarily leading to AF canting^5^.

Transport properties are not manifest in the algebraic theory but the CS approximation is equivalent to symmetry-constrained Hartree–Fock–Boboliubov (HFB) theory^25^,^33^, suggesting that SO(8) theory can be mapped onto Hartree–Fock (HF) transport calculations. We shall deal with the transport properties of the current theory in future papers.

Solutions depend on *G*_0_ and *b*_2_ in Eq. (14), which define effective interactions in the truncated space [highly renormalized relative to parameters in Eq. (1)]. They may be fixed by systematic comparison with data, enabling a robust prediction for the nature of the ground and other low-energy states. We expect modest impurity levels to modify the effective interaction parameters but leave dynamical symmetries intact.

The present ideas are similar to ones found in nuclear physics^20^ and high-*T*_c_ superconductors (SC)^28^,^29^, with all three cases exhibiting 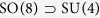 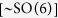 symmetry and a *critical dynamical symmetry* generalizing a quantum critical point to a *quantum critical phase* linking other phases through quantum fluctuations. These similarities may have implications for cross-disciplinary understanding of quantum phase transitions.

## Discussion

We have introduced an SO(8) model of monolayer graphene in a magnetic field that recovers SU(4) quantum Hall physics but implies new collective modes transcending explicitly-broken SU(4) that are leading candidates for the high-field ground state. Graphene SO(8) is isomorphic to a symmetry describing many complex nuclei and very similar to one describing high-*T*_c_ superconductors, suggesting a deep mathematical connection among these phenomena.

## Additional Information

**How to cite this article**: Wu, L.-A. and Guidry, M. The Ground State of Monolayer Graphene in a Strong Magnetic Field. *Sci. Rep.* **6**, 22423; doi: 10.1038/srep22423 (2016).
